# Towards optimizing the diagnosis of Lewy body dementia: Lessons from the NACC

**DOI:** 10.1002/alz.70794

**Published:** 2025-10-21

**Authors:** Anna E. Goodheart, Tess L. Brazier, Rong Ye, Patrick Stancu, Stephen N. Gomperts

**Affiliations:** ^1^ Harvard Medical School Boston Massachusetts USA; ^2^ Department of Neurology Massachusetts General Hospital Boston Massachusetts USA

**Keywords:** LBD, Lewy bodies, Lewy body dementia, NACC, National Alzheimer's Disease Coordinating Center

## Abstract

**INTRODUCTION:**

The diverse presentations and co‐pathologies of Lewy body dementia (LBD) present a diagnostic challenge. Utilizing the National Alzheimer's Coordinating Center (NACC) dataset, this study aimed to evaluate the concordance of premorbid diagnoses of LBD in individuals with autopsy‐confirmed neocortical Lewy body disease.

**METHODS:**

Demographics, clinical and neuropsychological presentations, Lewy body pathology, and Alzheimer's disease (AD) co‐pathology were related to clinical diagnosis.

**RESULTS:**

Diagnosis of LBD had high specificity but low sensitivity, with fewer than half of autopsy‐confirmed cases having received an LBD diagnosis. AD was the most common misdiagnosis. Participants diagnosed with AD were more likely to be female, to have a more amnestic phenotype, and to harbor a higher burden of AD co‐pathology, and were less likely to have documented clinical features characteristic of LBD.

**DISCUSSION:**

These results highlight the need to improve LBD diagnosis in research and clinical settings, a need that emerging biomarkers may help address.

**Highlights:**

- Less than half of cases with neocortical Lewy bodies were diagnosed with Lewy body dementia (LBD)
- Alzheimer's disease (AD) was the most common misdiagnosis
- Cases misdiagnosed were more likely to be female
- Cases diagnosed with AD presented with a more amnestic phenotype
- Cases diagnosed with AD had more AD co‐pathology

## 1 BACKGROUND

Lewy body dementia (LBD) is a common Alzheimer's disease (AD) and related dementia (ADRD) that is neuropathologically characterized by neuronal inclusions of misfolded α‐synuclein known as Lewy bodies.^1^, ^2^, ^3^ Clinically, LBD comprises two subtypes: dementia with Lewy bodies (DLB), in which the presenting phenotype is a dementing syndrome, and Parkinson's disease dementia (PDD), in which the development of cognitive impairment arises in the setting of PD.^2^, ^4^, ^5^ A clinical diagnosis of probable DLB is made in the setting of dementia with at least two of four core clinical features: parkinsonism (which, in the case of DLB, develops concurrently or after the onset of dementia), rapid eye movement (REM) sleep behavior disorder, visual hallucinations, and cognitive fluctuations. These features can commonly be found in PDD as well, although only parkinsonism (with onset at least 1 year prior to cognitive impairment) is necessary for a diagnosis of PDD.^4^, ^5^ In contrast to AD, LBD is more common in men than in women.^6^, ^7^ In addition, LBD tends to present with neuropsychological deficits in attention, executive function, and visuospatial processing, in contrast to the typical memory and language impairments predominant in AD.^8^, ^9^ However, the clinical and neuropsychological presentations of LBD can vary, presenting a diagnostic challenge. Furthermore, at least half of patients with LBD have neuropathological evidence of AD co‐pathology (amyloid beta [Aβ] and tau),^10^, ^11^, ^12^, ^13^, ^14^ which has the potential to further diversify clinical presentations. Accurate pre‐morbid diagnosis of neurodegenerative diseases including LBD and AD is imperative both clinically and for research purposes, particularly as clinical trials of disease‐modifying therapies are becoming increasingly prevalent.

The National Alzheimer's Coordinating Center (NACC) Uniform Data Set (UDS) is a large cohort of research participants with AD, ADRD, related neurological disorders, and healthy control participants.^15^, ^16^ The NACC UDS was established in 1999 and collates data from individuals evaluated at Alzheimer's Disease Research Centers (ADRCs) across the United States. ADRC participants are obtained from local communities and/or from ADRC‐affiliated medical centers. Participants are followed longitudinally and undergo detailed clinical characterization and neuropsychological testing, and, when possible, neuropathological confirmation of diagnosis on autopsy.

The objectives of this study were to perform an updated analysis on the sensitivity and specificity of the clinical diagnosis of LBD in the NACC cohort,^17^, ^18^ against the gold standard autopsy diagnosis of diffuse neocortical Lewy body deposition, to determine the clinical diagnoses in the misdiagnosed cases, and to explore differences in demographic features, clinical presentations, neuropsychological profiles, and co‐pathologies between accurately diagnosed and misdiagnosed participants. The overarching goal was to identify opportunities for improvement in the clinical diagnosis of LBD at ADRCs and more broadly for clinical and research purposes.

## 2 METHODS

### 2.1 Ethical approval and informed consent

Research using the NACC data is approved by the University of Washington Institutional Review Board and is conducted in accordance with the ethical standards outlined in the Declaration of Helsinki. Informed consent is obtained by the ADRCs.

### 2.2 NACC data

Data from 2005 through the September 2024 data freeze, comprising data from 46 ADRCs and three UDS versions, were used in this study. Access to NACC data is available upon reasonable request (https://naccdata.org/).

**RESEARCH IN CONTEXT**

1. **Systematic review**: The authors reviewed the literature using PubMed for publications relating to diagnosis of Lewy body dementia (LBD), particularly using the National Alzheimer's Disease Coordinating Center (NACC) data.
2. **Interpretation**: Our findings indicate that the diagnosis of LBD in the NACC cohort has high specificity but low sensitivity. LBD is often misdiagnosed as Alzheimer's disease.
3. **Future directions**: Our findings support the need for further improvements in the clinical diagnosis of Lewy body dementia and highlight the need for expanded use of α‐synuclein biomarkers for diagnostic purposes.

### 2.3 Neuropathologic and clinical diagnoses

Upon evaluation of a participant with cognitive impairment, ADRC clinicians are asked to make a judgement of the primary cause of the participant's cognitive impairment and, if relevant, any contributing causes. In the case of LBD, DLB and PDD can be distinguished by a separate question about the presence of PD. We used the diagnoses at the last clinical visit (that closest to autopsy) for analysis. Autopsy findings of neocortical Lewy bodies were used to define a neuropathological diagnosis of LBD. Premorbid primary and contributing diagnoses of autopsy‐confirmed LBD cases were determined. Sensitivity and specificity of a clinician diagnosis of LBD based on the gold standard neuropathologic diagnoses were calculated. A true‐positive (correct) diagnosis was defined as a diagnosis of primary or contributing LBD. A false‐negative diagnosis (misdiagnosis) was defined as diagnosis other than primary or contributing LBD.

### 2.4 Demographic groups

Using a two‐tailed *t*‐test, age at last visit was compared between the group correctly diagnosed with primary or contributing LBD and the group misdiagnosed. Sex was compared between the groups using a chi‐square test.

### 2.5 Clinical features

Because AD comprised the majority of misdiagnosed cases, the clinical features of those misdiagnosed with AD versus those diagnosed correctly with LBD were examined further. The documented presence of core clinical features (parkinsonism, REM sleep behavior disorder, visual hallucinations, and cognitive fluctuations) were compared among three groups: a premorbid diagnosis of primary LBD, a premorbid diagnosis of primary AD with contributing LBD, and a premorbid diagnosis of primary AD without contributing LBD. A clinical feature was determined to be present in an individual if it was marked as present at any study visit. Because these clinical features were not asked consistently on every version of the UDS, an individual with no entries for a particular clinical feature was excluded from the analysis of that clinical feature. The presence of clinical features was related to the diagnoses using pairwise chi‐square tests with Bonferroni correction for multiple comparisons.

### 2.6 Neuropsychologic battery test scores

Presenting (first available) neuropsychological battery test scores were analyzed. Only neuropsychological tests that were conducted in all UDS versions^19^ were used. The following neuropsychological tests representing core domains of cognitive impairment were used: Mini‐Mental State Examination (MMSE) (global cognition), Logical Memory IIA Delayed Recall (memory), Boston Naming Test (language), Trail Making Test Part A (Trails A; attention), and Trail Making Test Part B (Trails B; executive functioning). As there is no targeted test of visuospatial functioning available in the early UDS versions, this cognitive domain was not independently examined, while recognizing that visuospatial functioning is a component of both Trails tests. Presenting neuropsychological battery test scores among the three groups (diagnosis of primary LBD, diagnosis of primary AD with contributing LBD, and diagnosis of primary AD without contributing LBD) were compared using general linear models with Tukey post hoc testing.

### 2.7 Co‐pathologies

The degree of Alzheimer's co‐pathology, defined by Consortium to Establish a Registry for Alzheimer's Disease (CERAD)^20^ stage of amyloid plaques and Braak stage^21^ of tau tangles, were compared in the LBD diagnosis group versus the AD diagnosis groups using general linear models with Tukey post hoc testing. Thal phase was not evaluated, as it was available only for autopsies after 2014.

The severity of substantia nigra (SN) neuron loss, available in more recent versions of the neuropathologic reports (versions 10 [introduced in 2014] and later) was also examined. SN neuron loss is coded as “none, mild, moderate, or severe.” We compared these severity scores among diagnostic groups using an analogous model to the Alzheimer's co‐pathology models. The categories of SN neuron loss severity in participants with and without clinically recognized parkinsonism were compared using a chi‐square test. SAS statistical software version 9.4 was used for statistical analyses.

## 3 RESULTS

### 3.1 Clinical diagnoses

Out of 8188 available autopsy cases, 1020 had neocortical Lewy bodies. Notably, only 419 (41%) of these 1020 cases were diagnosed with primary or contributing LBD in life (true‐positive diagnosis). That number dropped to 330 (32%) with a diagnosis of primary LBD. Eighty‐two cases (8%) of the 1020 cases were diagnosed with primary AD with contributing LBD. A breakdown of LBD diagnoses by cognitive status and by DLB versus PDD is presented in Table S1.

Of the 601 cases not diagnosed with primary or contributing LBD (false‐negative diagnoses/misdiagnoses), 483 (80%) were diagnosed with primary AD without contributing LBD, making up 47% of the total 1020 autopsy cases with neocortical Lewy bodies. Common diagnoses of the remaining neocortical Lewy body cases not diagnosed with LBD or AD included normal cognition (*n* = 33), an atypical parkinsonian disorder (*n* = 20), vascular dementia (*n *= 14), and frontotemporal dementia (*n *= 13).

### 3.2 Sensitivity and specificity

A total of 857 of all 8188 autopsied cases had a clinical diagnosis of primary or secondary LBD, giving a sensitivity and specificity of a diagnosis of primary or contributing LBD of 41% (95% confidence interval [CI] 38–44) and 94% (95% CI 93–94), respectively.

Although sensitivities remained similarly low, with CIs overlapping global sensitivities, there was a trend toward improving specificity in later years, corresponding with the 2015 release of UDS version 3 (and the accompanying Lewy Body Dementia Module) as well as with the publication of the most updated diagnostic criteria for DLB in 2017^5^ (Tables S2 and S3).

### 3.3 Demographic features

False‐negative diagnoses were more likely than true‐positive diagnosis to be in female patients (false negatives 46% female, true positives 23% female, *p* < 0.0001). Misdiagnosed cases were also slightly older (false‐negative mean (+ SD) age 78 ± 11 years, true‐positive mean age 77 ± 8 years), although this age difference did not reach statistical significance.

### 3.4 Clinical features

**FIGURE 1:**
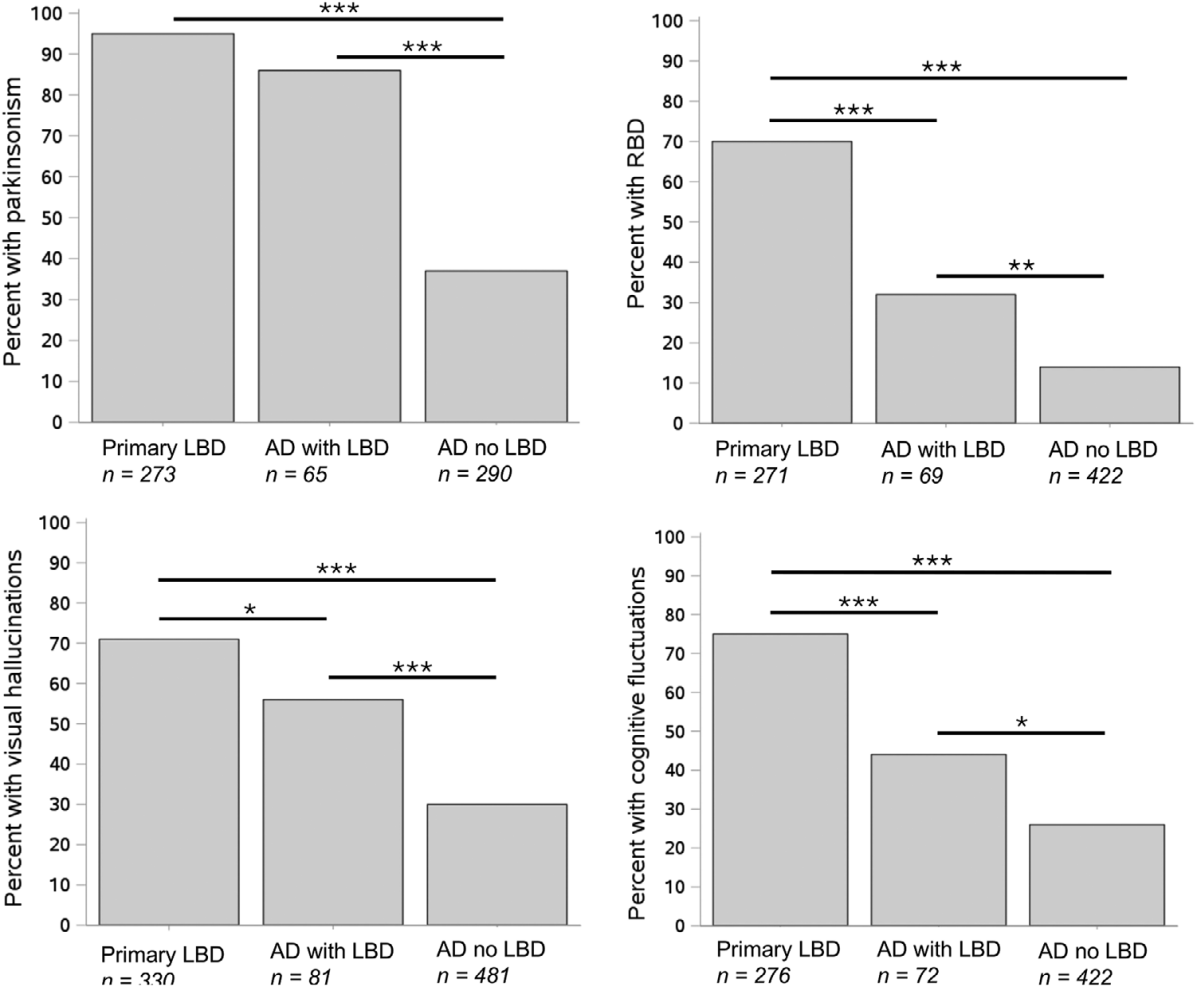
Clinical characteristics of participants with autopsy‐confirmed neocortical Lewy body disease: The percentage of cases with each clinical characteristic is shown for three diagnostic groups: primary Lewy body dementia (LBD), primary Alzheimer's disease (AD) with contributing LBD (AD with LBD), and primary AD without contributing LBD (AD no LBD). Numbers of cases with available data for each test are indicated. * denotes statistically significant difference in diagnosis based on presence or absence of the clinical characteristic (* *p *< 0.0167 [Bonferroni adjusted *p*‐value], ** *p* < 0.001 *** *p* < 0.0001).

Because a substantial number of cases with Lewy body pathology were diagnosed with primary AD, we focused further analyses on this group, separating them into diagnoses of primary AD with contributing LBD (*n *= 82) and primary AD without contributing LBD (*n* = 483) and comparing clinical characteristics of these groups with primary LBD diagnosis (*n* = 330). Participants with autopsy‐confirmed neocortical Lewy body disease who were either diagnosed with primary LBD or diagnosed with AD with contributing LBD were more likely to have been identified as having clinical features common in LBD, namely parkinsonism, REM sleep behavior disorder, visual hallucinations, and cognitive fluctuations, compared to participants with autopsy‐confirmed neocortical Lewy body disease who were diagnosed with AD without contributing LBD (Figure 1).

### 3.5 Neuropsychological battery test results

**FIGURE 2:**
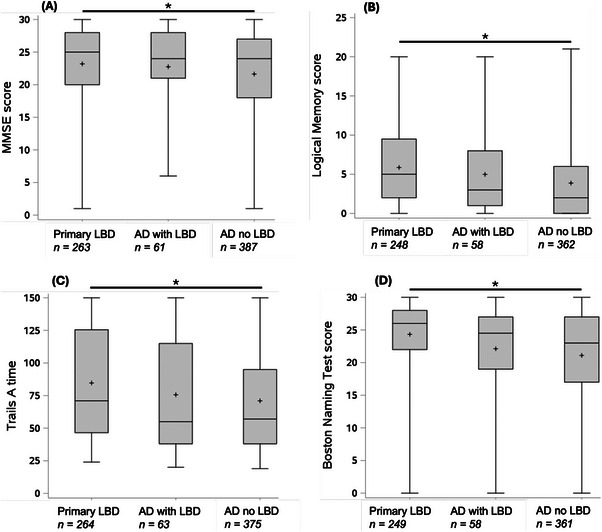
Neuropsychological presentations of participants with autopsy‐confirmed neocortical Lewy body disease: Neuropsychological battery test scores in participants with autopsy‐confirmed neocortical Lewy body pathology who were diagnosed with either primary Lewy body dementia (LBD), primary Alzheimer's disease (AD) with contributing LBD (AD with LBD), or primary AD without contributing LBD (AD no LBD) are shown. (A) MMSE (Mini‐Mental State Examination) reflects global cognition (of 30, lower score worse). (B) Logical Memory IIA Delayed Recall evaluates the memory domain (of 25, lower score worse). (C) Boston Naming Test evaluates the language domain (of 30, lower score worse). (D) Trail Making Test Part A (Trails A) evaluates attention (number of seconds taken to complete, up to 150, higher score worse). Numbers of cases with available data for each test are indicated. Box represents median and interquartile range; + represents mean, whiskers represent minimum and maximum. *p < 0.05 difference between the primary LBD and AD no LBD group.

Participants misdiagnosed as primary AD without contributing LBD were, compared to those diagnosed with primary LBD, more likely to present with greater global cognitive deficits (lower score on the MMSE), memory deficits (lower score on the Logical Memory Delayed Recall test), and language deficits (lower score on the Boston Naming Test). Participants diagnosed with primary LBD were more likely to present with attentional deficits (longer time to complete the Trails A task). There was no significant difference among groups in executive function (Trails B task). There were no significant differences in neuropsychological test results between the group diagnosed with primary AD with contributing LBD and either of the other groups: those with primary LBD or those with primary AD without contributing LBD (Figure 2).

### 3.6 Co‐pathologies

**FIGURE 3:**
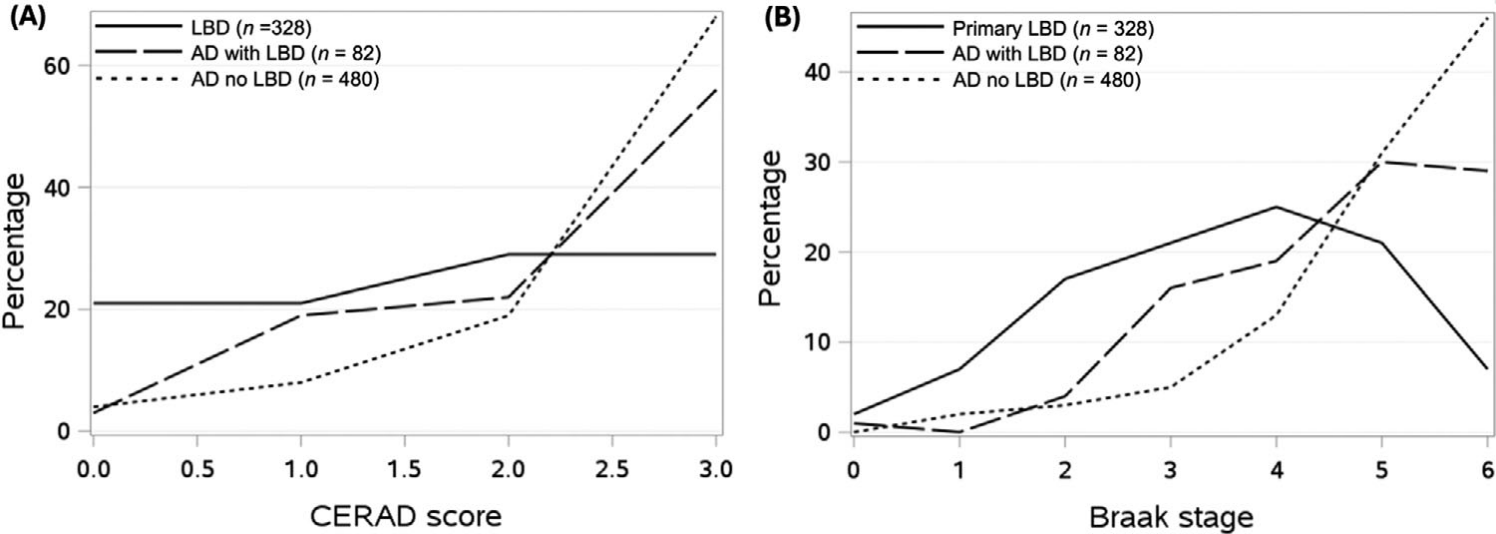
Alzheimer's co‐pathology of participants with autopsy‐confirmed neocortical Lewy body disease: Percentage of cases with (A) CERAD scores of amyloid co‐pathology and (B) Braak stages of tau co‐pathology for the following diagnoses: primary Lewy body dementia (LBD), primary Alzheimer's disease (AD) with contributing LBD (AD with LBD), and AD without contributing LBD (AD no LBD)).

Participants with autopsy‐confirmed neocortical Lewy bodies who had a clinical diagnosis of primary LBD had a lower mean CERAD score (mean 1.7 ± 1.1) compared to participants clinically diagnosed with primary AD with contributing LBD (mean 2.3 ± 0.9) (*p *< 0.0001) and compared to participants clinically diagnosed with primary AD without contributing LBD (mean 2.5 ± 0.8) (*p *< 0.0001; Figure 3a). There was no statistically significant difference in CERAD scores between participants diagnosed with AD with contributing LBD and AD without contributing LBD.

Participants with autopsy‐confirmed neocortical Lewy bodies who had a clinical diagnosis of primary LBD had a lower Braak stage (mean 3.5 ± 1.5) compared to participants clinically diagnosed with primary AD with contributing LBD (mean 4.6 ± 1.3) (*p *< 0.0001) and compared to participants clinically diagnosed with primary AD without contributing LBD (mean 5.0 ± 1.3) (*p *< 0.0001). Participants with a clinical diagnosis of primary AD with contributing LBD had a lower Braak stage (mean 4.6 ± 1.3) compared to primary AD without contributing LBD (mean 5.0 ± 1.3) (*p* = 0.0116; Figure 3b).

Approximately half (*n *= 521) of the cases of autopsy‐confirmed neocortical Lewy body disease had neuropathologic data on severity of SN neuron loss available. Within this group, participants who had a clinical diagnosis of primary LBD had a higher SN loss severity score (mean 2.3 ± 0.8) compared to participants clinically diagnosed with primary AD with contributing LBD (mean 1.9 ± 0.9) (*p *= 0.0064) and compared to participants clinically diagnosed with primary AD without contributing LBD (mean 1.6 ± 0.9) (*p* = < 0.0001). The difference in SN neuron loss between groups diagnosed with AD with contributing LBD and those diagnosed with AD without contributing LBD was not statistically significant. Participants who were clinically recognized as having parkinsonism had more severe SN neuron loss compared to those not clinically recognized as having parkinsonism (*p *< 0.0001).

## 4 DISCUSSION

The diverse presentations of LBD and frequent co‐occurrence of AD co‐pathology present a diagnostic challenge. To assess the accuracy of the clinical diagnosis of LBD in the NACC cohort, we evaluated premorbid clinical diagnoses of autopsy‐confirmed cases with neocortical Lewy body pathology. Our findings provide actionable information for clinicians when evaluating research participants and patients.

Our observation of high specificity but low sensitivity of the diagnosis of LBD in the NACC cohort is consistent with prior findings in older versions of the NACC dataset^17^, ^18^ and build upon previous reports by examining the characteristics of individuals with true‐positive and false‐negative diagnoses. The high specificity indicates that the LBD cohort in the NACC dataset is robust for the study of LBD. However, many cases of LBD are being missed, restricting the size of the LBD cohort and also diluting the AD cohort with unrecognized LBD cases.

Although there was a small trend toward improved specificity in later years, there were not substantial improvements in sensitivity or specificity with introduction of updated forms of the UDS, the introduction of the Lewy Body Dementia Module in 2015, or the publication of updated Consensus Criteria for DLB in 2017.^5^ The Lewy Body Dementia Module provides an in‐depth assessment of clinical symptoms and signs commonly found in LBD, including detailed assessments of cognitive fluctuations, sleep, and neuropsychiatric symptoms. The addition of this Module has great value for the study of LBD using the NACC cohort. However, the Lewy Body Dementia Module has no bearing on overall sensitivity because it is administered only to select participants identified as at risk for LBD, rather than being used universally. Our findings highlight the need for LBD diagnostic criteria to be outlined and considered during the UDS assessment itself.

Our finding that LBD cases misdiagnosed with AD were more likely to be female has several implications. First, it raises the possibility that misdiagnosis in women may contribute to the observation that LBD is more common in men.^6^, ^7^ In addition, our findings support the possibility that LBD may present differently in women than in men and specifically that it may present more like AD, consistent with prior reports showing that women with autopsy‐confirmed Lewy body pathology tend to be older, less likely to experience visual hallucinations, and have more tau pathology on autopsy.^22^, ^23^

In this study, we also observed a reduced percentage of clinical features of Lewy body disease in the misdiagnosed participants, either secondary to the absence of these features or the absence of clinician recognition or documentation of these features. To enhance the detection of these clinical features and improve the diagnostic accuracy of LBD in the NACC cohort, the integration of a more detailed checklist of LBD clinical features into the UDS may be useful for clinician diagnosis. Consideration of LBD clinical features across visits is also of value, as some features may evolve over time or improve with pharmaceutical interventions.

It is well‐established that patients with LBD tend to have a neuropsychological presentation of inattention, executive dysfunction, and visuospatial processing deficits, in contrast to AD, in which memory and language impairments tend to predominate at presentation.^8^, ^9^ Even so, in this cohort, many participants with autopsy‐confirmed neocortical Lewy body disease presented with memory and language impairments. The fact that these cases were common and were more likely to be misdiagnosed with AD suggest that the presence of prominent memory or language deficits does not refute a diagnosis of LBD.

Strengths of this study include the large number (greater than 1000) of autopsies with neocortical Lewy body pathology, the longitudinal clinical characterization and detailed neuropsychological profiling of participants in the NACC cohort, as well as its geographic diversity across the United States. However, there are several limitations as well, including the limited racial, ethnic, and educational diversity of the cohort.^24^, ^25^ Another limitation is the change in the method of recording Lewy body neuropathology over time: The distinction between limbic and amygdala‐predominant Lewy body disease is a more recent addition (2014) to the neuropathologic reports, whereas earlier autopsy reports combined the two. Because isolated amygdala‐predominant Lewy bodies can be seen in AD^5^ and are not necessarily indicative of a diagnosis of Lewy body dementia, this is a critical distinction. To deal with this issue, we included only autopsy cases with neocortical Lewy bodies, and future work that includes participants with limbic‐predominant disease will be useful. Another limitation, inherent to autopsy data, is the possibility of the development of the pathology in question in the interim between the last clinical evaluation and death. Other limitations include the absence of questions about parkinsonism, REM sleep behavior disorder, and cognitive fluctuations on the Clinical Judgement of Symptoms form in UDS version 1, and the substantial number of participants missing an answer to the question about parkinsonism in all UDS versions, which limited the sample size available to analyze this clinical feature. In addition, the absence of dedicated visuospatial function testing in earlier UDS versions precluded a comprehensive assessment of visuospatial dysfunction, a cognitive domain commonly impacted in LBD.^2^ Despite these limitations, we were still able to demonstrate key differences in true‐positive and false‐negative diagnoses of LBD.

Taken together, our findings demonstrate that although the specificity of the clinical criteria for LBD in the NACC cohort is remarkably high, missed diagnoses are common, especially among women. The recent advent of α‐synuclein biomarkers in cerebrospinal fluid and skin^26^, ^27^ provides a means to improve sensitivity. The integration of such methods into research cohorts and clinical care has great promise to improve the diagnostic accuracy of LBD with and without AD co‐pathology. This approach is likely to prove useful also in clinical trials of disease‐modifying therapies.

## CONFLICT OF INTEREST STATEMENT

The authors declare no conflicts of interest. Author disclosures are available in the Supporting Information.

## CONSENT STATEMENT

All human subjects provided written informed consent.

## Supporting information



Supporting Information

Supporting Information
