# Supplementary material for: Towards optimizing the diagnosis of Lewy body dementia: Lessons from the NACC
Source: Alzheimers Dement. 2025 Oct 21;21(10):e70794. doi: 10.1002/alz.70794 (PMC12538637; doi:10.1002/alz.70794)
Supplement: Supplementary file 1 — Supporting Information [file ALZ-21-e70794-s001.docx]

**Supplementary Material**

| **Cognitive status of primary or contributing LBD** | ***n* = 419** |
| --- | --- |
| Dementia | *n* = 394 (94%) |
| Mild cognitive impairment | *n* = 21 (5%) |
| **Diagnosis of primary LBD** | ***n* = 330** |
| Dementia with Lewy bodies | *n* = 231 (70%) |
| Parkinson’s disease dementia | *n* = 99 (30%) |

**Supplementary Table 1:** Breakdown of cognitive status and clinical diagnoses of individuals with neocortical Lewy bodies who were diagnosed with LBD during life. LBD = Lewy body dementia.

| **UDS Version 1 (2005)** | ***n* = 1,117** |
| --- | --- |
| Sensitivity (95% CI) | 46% (38 - 55) |
| Specificity (95% CI) | 93% (92 - 95) |
| **UDS Version 2 (2008)** | ***n* = 4,010** |
| Sensitivity (95% CI) | 41% (37 - 46) |
| Specificity (95% CI) | 93% (92 - 94) |
| **UDS Version 3 (2015)*** | ***n* = 3,061** |
| Sensitivity (95% CI) | 39% (34 - 44) |
| Specificity (95% CI) | 95% (95 - 96) |

**Supplementary Table 2:** Sensitivities and specificities of a diagnosis of LBD across Uniform Data Set (UDS) versions. *The Lewy Body Dementia Module was introduced alongside UDS version 3.

| **Pre-2017** | ***n* = 6,552** |
| --- | --- |
| Sensitivity (95% CI) | 41% (37 - 44) |
| Specificity (95% CI) | 93% (93 - 94) |
| **Post-2017** | ***n* = 1,636** |
| Sensitivity (95% CI) | 42% (35 - 49) |
| Specificity (95% CI) | 96% (95- 97) |

**Supplementary Table 3:** Sensitivities and specificities of a diagnosis of LBD pre- and post- publication of the 2017 Fourth Consensus Criteria for DLB diagnosis.
